# Quantum-confined superfluid reactions

**DOI:** 10.1039/d0sc03574b

**Published:** 2020-08-26

**Authors:** Yuwei Hao, Shuai Pang, Xiqi Zhang, Lei Jiang

**Affiliations:** Key Laboratory of Bio-Inspired Smart Interfacial Science and Technology of Ministry of Education, School of Chemistry, Beijing Advanced Innovation Center for Biomedical Engineering, Beihang University Beijing 100191 P. R. China jianglei@iccas.ac.cn; CAS Key Laboratory of Bio-Inspired Materials and Interfacial Science, Technical Institute of Physics and Chemistry, Chinese Academy of Sciences Beijing 100190 P. R. China xqzhang@mail.ipc.ac.cn; School of Future Technology, University of Chinese Academy of Sciences Beijing 100049 P. R. China

## Abstract

A helium atom superfluid was originally discovered by Kapitsa and Allen. Biological channels in such a fluid allow ultrafast molecule and ion transport, defined as a quantum-confined superfluid (QSF). In the process of enzymatic biosynthesis, unique performances can be achieved with high flux, 100% selectivity and low reaction activation energy at room temperature, under atmospheric pressure in an aqueous environment. Such reactions are considered as QSF reactions. In this perspective, we introduce the concept of QSF reactions in artificial systems. Through designing the channel size at the van der Waals equilibrium distance (*r*_0_) for molecules or the Debye length (*λ*_D_) for ions, and arranging the reactants orderly in the channel to satisfy symmetry-matching principles, QSF reactions in artificial systems can be realized with high flux, 100% selectivity and low reaction activation energy. Several types of QSF-like molecular reactions are summarized, including quantum-confined polymerizations, quasi-superfluid-based reactions and superfluid-based molecular reactions, followed by the discussion of QSF ion redox reactions. We envision in the future that chemical engineering, based on multi-step QSF reactions, and a tubular reactor with continuous nanochannel membranes taking advantage of high flux, high selectivity and low energy consumption, will replace the traditional tower reactor, and bring revolutionary technology to both chemistry and chemical engineering.

## Abstract 2

The concept of quantum-confined superfluid reactions is introduced into artificial systems, which is expected to be useful in future chemical engineering.

## Graphical abstract

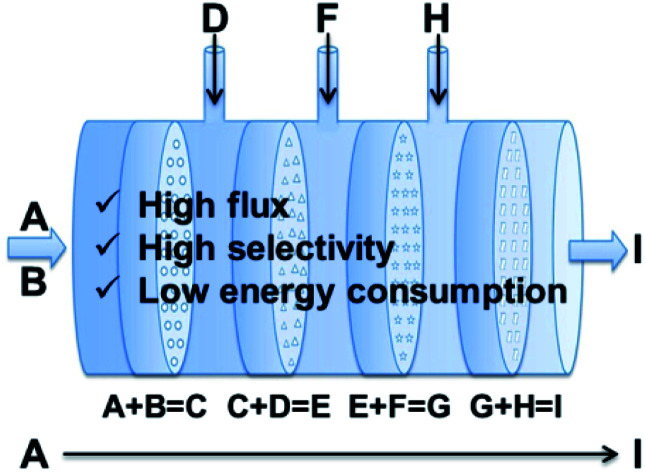

## 1. Introduction

**Fig. 1:**
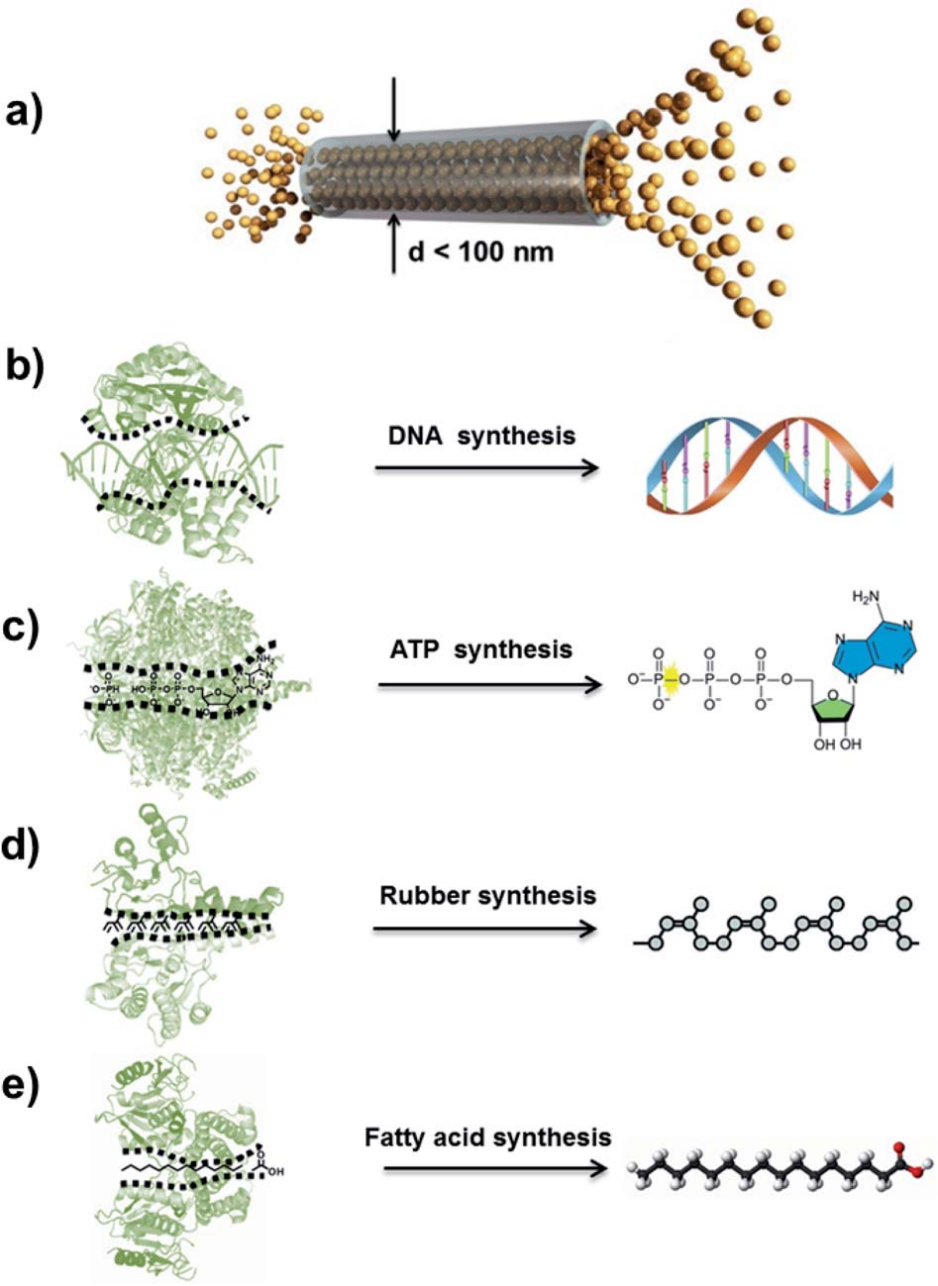
Atomic superfluid and QSF reactions in a biological system. (a) Schematic representation of ^4^He superfluid transport across a channel with a diameter less than 100 nm, indicating highly ordered ^4^He molecules stacking and transport. Reproduced with permission from ref. 7. Copyright 2019, Wiley-VCH. (b) The DNA double helix can be polymerized with a remarkably precise sequence and structure in the DNA replication process. (c) During the preparation of ATP from ADP and phosphate, ATP synthase catalyzes the reaction with low energy consumption. (d) Biosynthesis of natural rubber catalyzed by rubber transferase, where the rubber molecules grow *via* living polymerization with the continuous addition of monomers. (e) Fatty acids are synthesized *via* a series of decarboxylative Claisen condensation reactions by fatty acid synthase. These enzymatic reactions realize QSF reactions with high flux, 100% selectivity and low reaction activation energy at room temperature, under atmospheric pressure in an aqueous environment.

The concept of an atomic superfluid can be traced back to the 1930s, when Kapitsa and Allen *et al.* observed a ^4^He fluid below 2.17 K.^1,2^ The nearly zero viscosity of the ^4^He superfluid means that there is no loss of kinetic energy, and its velocity through capillaries with varying diameters increases rapidly as its channel diameter decreases.^3^ When the intrinsic diameter of a capillary is below 100 nm, the velocity of ^4^He only depends on the temperature rather than pressure and channel length (Fig. 1a).^4,5^ In biological ion or molecule channels, unique characteristics of ultrafast transport, high flux and low energy loss can be realized, with such phenomena being defined as a quantum-confined superfluid (QSF).^6,7^ In the process of enzymatic biosynthesis, the reactant molecules arrange in the channels orderly, which greatly reduces the reaction activation energy, realizing 100% selectivity and ultrahigh flux in the bodily environment. Such reactions are considered as QSF reactions,^8,9^ such as DNA, ATP, natural rubber and fatty acid syntheses. For example, four kinds of deoxynucleotides are individually inserted into complementary strands in the DNA replication process with accurate positions and conformation due to base-pairing rules (Fig. 1b).^10^ Thus, the desired DNA double helix can be polymerized with remarkably precise sequence and structure. During the preparation of ATP from ADP and phosphate, the ATP synthase catalyses the reaction with low energy consumption (Fig. 1c).^11,12^ In the biosynthesis of natural rubber, the monomer of isopentenyl pyrophosphate is catalysed *in vivo* by rubber transferase, and the rubber molecules grow *via* “living carbocationic polymerization” with the continuous addition of monomers (Fig. 1d).^13^ It is noteworthy that specific rubber transferases can regulate *cis*–*trans* stereo-regulation, demonstrating symmetry-matching principles during the reaction process. The fatty acids are synthesized *via* a series of decarboxylative Claisen condensation reactions by fatty acid synthase, and the growing fatty acid chain is carried between these active sites of synthase (Fig. 1e).^14^ These enzymatic reactions involve the highly-ordered arrangement of reactants in a confined nanospace, realizing QSF reactions with high flux, 100% selectivity and low reaction activation energy at room temperature, under atmospheric pressure in an aqueous environment.

In this perspective, we introduce the concept of QSF reactions in artificial systems. Quantum-confined molecule superfluids and QSF-like molecule reactions, including quantum-confined polymerizations, quasi-superfluid-based reactions and superfluid-based molecule reactions are summarized. Then quantum-confined ion superfluids and QSF ion redox reactions are discussed. We further look forward to the future chemical engineering based on QSF reactions, which would achieve excellent performances in terms of high flux, high selectivity and low energy consumption.

## 2. Quantum-confined molecule superfluids and QSF-like molecule reactions

**Fig. 2:**
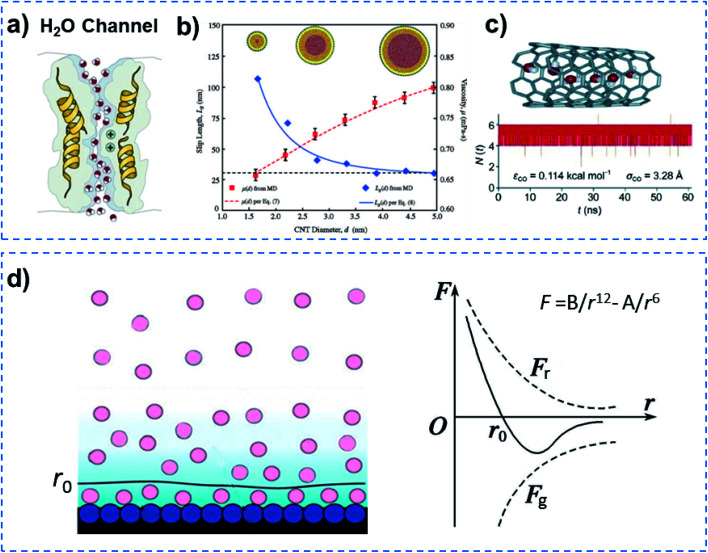
Quantum-confined molecule superfluid. (a) Biological water channel with an ultrahigh water flux comprising an ordered water strand, indicating a quantum method of transport. (b) The flow rate significantly increases with decreasing diameter of the CNT channel, when the diameter is reduced to 1.66 nm, an ordered water strand is formed. Reproduced with permission from ref. 19. Copyright 2008, ACS. (c) MD simulation of a single molecular water strand inside a carbon nanotube and the number of water molecules inside the nanotube as a function of time. Reproduced with permission from ref. 20. Copyright 2001, Nature Publishing Group. (d) Schematic of a multi-molecule adsorption layer (left) and molecular force curve (right). A quantum-confined molecule superfluid can be formed by decreasing the channel size to a distance of the van der Waals equilibrium distance (*r*_0_) for the molecule. Quantum-confined molecule superfluid either in biological or artificial channels show unique performances in terms of high flux and low energy consumption.

Quantum-confined molecule superfluids can be found in both biological and artificial systems when the channel size is reduced to the distance of the van der Waals equilibrium distance (*r*_0_) of molecules. Biological water channels with ultrahigh water flux comprise ordered water strands, indicating quantum method of transport (Fig. 2a). During the past decade, researchers found that the ultrafast water flow through an aligned carbon nanotube (CNT) membrane is 4–5 orders of magnitude higher than that predicted from conventional fluid-flow theory.^15,16^ Both experimental results and simulations have demonstrated that the confined water flux can increase up to seven orders of magnitude in the hydrophobic nanochannels compared to that of bulk water.^17^ In the case of molecular dynamics (MD) simulations, water orientations and motions confined in nanochannels suggest that water molecules can arrange spontaneously in an ordered way, when the diameter of the CNT channel is below 8.6 Å. But in the wider CNT channel, water molecules are disordered just as in bulk water.^18^ Another MD simulation for water transport in CNTs with different diameters (1.66–4.99 nm) demonstrated that the enhancement of water flow velocity increased from 47 to 433, when the diameter of the CNT channel decreased (Fig. 2b).^19^ Hummer and co-workers reported spontaneous and continuous filling of a one-dimensionally ordered chain of water molecules (about five water molecules) in a hydrophobic CNT, which transited pulse-like through the nanotube (Fig. 2c).^20^ It is noteworthy that they ignored the fact that the inlet of a CNT is hydrophilic,^15^ although no influence on their results was observed. Our previous simulation showed that water can only penetrate into a nanotube with a hydrophilic inlet.^21^Fig. 2d shows the formation process of a quantum-confined molecule superfluid by decreasing the channel size to the distance of the van der Waals equilibrium distance (*r*_0_) of the molecules. Therefore, a quantum-confined molecule superfluid, either in biological or artificial channels, shows unique performances, with high flux and low energy consumption.

**Fig. 3:**
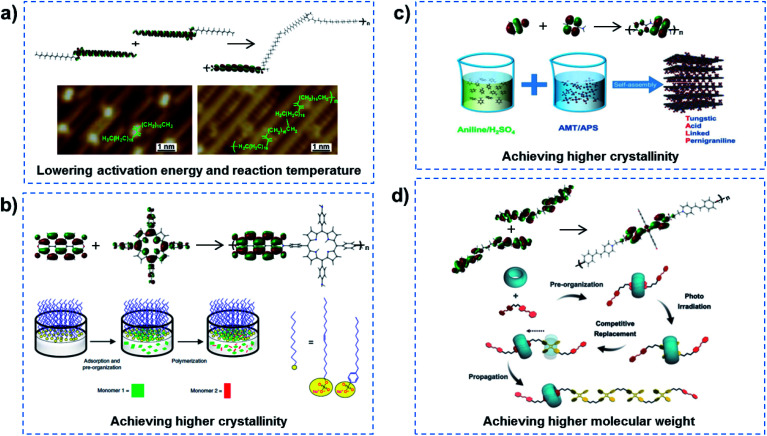
Quantum-confined polymerization. (a) Linear alkanes adsorbed in Au (1 × 3)-(110), preassembled and packed closely in the 1D atomic channels along the (110) lattice direction, indicating a polymerization process with lowered activation energy and reaction temperature. Reproduced with permission from ref. 22. Copyright 2018, ACS. (b) Controlled synthesis of 2D polyimide crystals assisted by surfactant monolayers, achieving higher crystallinity of the polymer. Reproduced with permission from ref. 23. Copyright 2019, Nature Publishing Group. (c) 2D conductive hybrid lamella with higher crystallinity and electrical conductivity for intercalative charge storage have been fabricated in a 2D confined space. Reproduced with permission from ref. 24. Copyright 2018, Wiley-VCH. (d) Supramolecular catalyzed polymerization in a CB[8] channel can flip along and elongate the polymer chain, leading to the obtaining of a high molecular weight polymer. Reproduced with permission from ref. 25. Copyright 2018, Wiley-VCH. In quantum-confined polymerization reactions, the reactant molecules are orderly arranged in the channel to satisfy the symmetry-matching principles of frontier molecular orbital theory, achieving reactions with low activation energy.

In order to realize QSF molecule reactions in an artificial system, the reactants should be arranged orderly in the channel to satisfy symmetry-matching principles, and the channel size should be designed at a distance of the van der Waals equilibrium distance (*r*_0_) of the molecules, then QSF reactions can be realized with high performance, including high flux, 100% selectivity and low reaction activation energy. Arrangement of the reactant molecules orderly in the channel to satisfy symmetry-matching principles can realize a low reaction activation energy of the reactions, and such reactions are considered to be quantum-confined reactions. Specific Au(110) surfaces can serve as a confined space and efficient catalysts to lower the energy barriers, resulting in high-activity and selective reaction processes. Linear 18,19-dimethylidenehexatriacontane (DMH) alkanes adsorbed in Au (1 × 3)-(110) have been shown to preassemble and pack closely in the 1D atomic channels along the (110) lattice direction, and then undergo polymerization with a low activation energy and reaction temperature (Fig. 3a).^22^ Moreover, controlled synthesis of few-layer 2D polyimide crystals on the surface of water (air–water interface) was proved through the reaction between amine and anhydride monomers, assisted by surfactant monolayers, which resulted in polymers with high crystallinity and a thickness of ∼2 nm (Fig. 3b).^23^ The formation of crystalline polymers was attributed to the pre-organization of monomers at the water–surfactant interface. Similarly, 2D conductive hybrid lamella with high crystallinity and electrical conductivity for intercalative charge storage have been fabricated in a 2D confined space (Fig. 3c).^24^ Another example is supramolecular catalyzed polymerization, where cucurbit[8]uril (CB[8]) can flip along and elongate the polymer chain as a supramolecular confinement agent and catalyst for the fabrication of covalent polymers under light irradiation, while the molecular weights of the obtained polymer could be increased by controlling the irradiation time or the monomer (Fig. 3d).^25^ Similar supramolecular polymerization was reported to construct a NIR-II chromophore *via* the tailor-made assembly of organic radicals for photothermal conversion and therapy.^26^ In addition, porous coordination polymers with nanochannels and basic interaction sites allowed the highly accelerated, stereocontrolled, and monomerselective polymerization of substituted acetylenes.^27^ Therefore, quantum-confined polymerizations can achieve low reaction activation energy of the reactions.

**Fig. 4:**
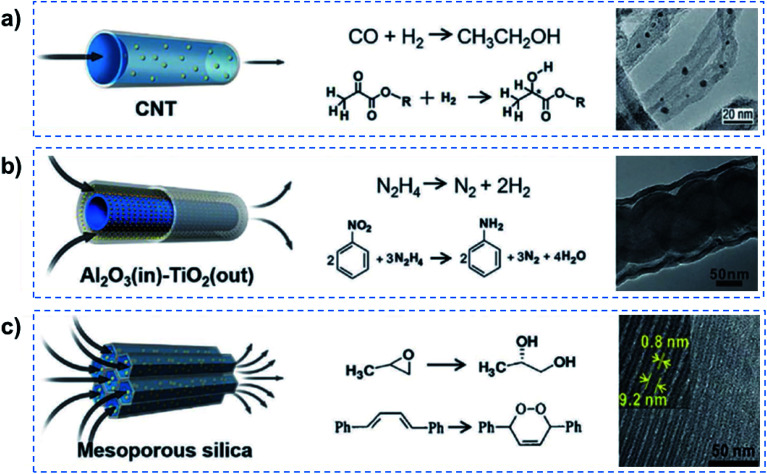
Quasi-superfluid-based chemical reactions. (a) Nanoconfined catalytic reaction in 1D CNT nanochannels with catalyst nanoparticles inside, exhibiting higher reaction yield and enantioselectivity. (b) Nitrobenzene hydrogenation confined within 1D tubular nanochannels achieves a remarkably high catalytic efficiency and 99% selectivity of aniline. (c) The confined catalysts in mesoporous silica nanochannels provide excellent activity and selectivity for the hydrolytic kinetic resolution of epoxides and photosensitized oxidation of alkenes. Reproduced with permission from ref. 8. Copyright 2019, Wiley-VCH. Quasi-superfluid-based reactions performed in nanoconfined channels exhibit enhanced performances in terms of high selectivity and low reaction activation energy.

Some previous studies have successfully realized quasi-superfluid-based reactions with improved performance.^8^ For example, Rh-based particles were confined inside CNTs to catalyze Fischer–Tropsch synthesis, and enhanced catalytic activity was achieved for the conversion of syngas to ethanol (Fig. 4a). The overall rate of ethanol yield inside the CNTs was higher by one order of magnitude than that outside the nanotubes even though the outer surface was more accessible. Chirally modified Pt catalysts were designed inside the CNTs, which could realize relatively higher enantioselectivity and activity than that loaded outside the CNTs for the asymmetric hydrogenation of α-ketoesters.^28^ The enhancement was attributed to the nanoconfinement and enrichment of the Pt nanocatalyst with the chiral modifier cinchonidine inside CNT channels. Besides this, tandem hydrogenation reactions, such as nitrobenzene hydrogenation using hydrazine exhibited high catalytic efficiency and 99% selectivity of aniline, resulting from the catalysis of tube-in-tube Al_2_O_3_/Ni–Pt/TiO_2_ consisting of an inside Ni/Al_2_O_3_ interface and outside Pt/TiO_2_ interface (Fig. 4b).^29^ When encapsulated in the nanochannels of SBA-15, SBA-16, MCM-41, the Co(iii) complex catalysts reached 98% enantioselectivity and outstanding reusability in the hydrolysis of propylene oxide (Fig. 4c).^30^ In a confined photosensitized oxidation system, alkenes were confined in the channels of Na-ZSM-5 zeolites, and the products were obtained selectively from singlet oxygen oxidation.^31^ More recently, a heterogeneous catalyst system, consisting of AuPd alloy nanoparticles fixed within aluminosilicate zeolite crystals, was reported for enhanced methanol productivity in methane oxidation by *in situ* generated hydrogen peroxide at mild temperature (70 °C).^32^ It can be concluded that these quasi-superfluid-based reactions performed in nanoconfined channels exhibited enhanced performances in terms of high selectivity and low reaction activation energy.

**Fig. 5:**
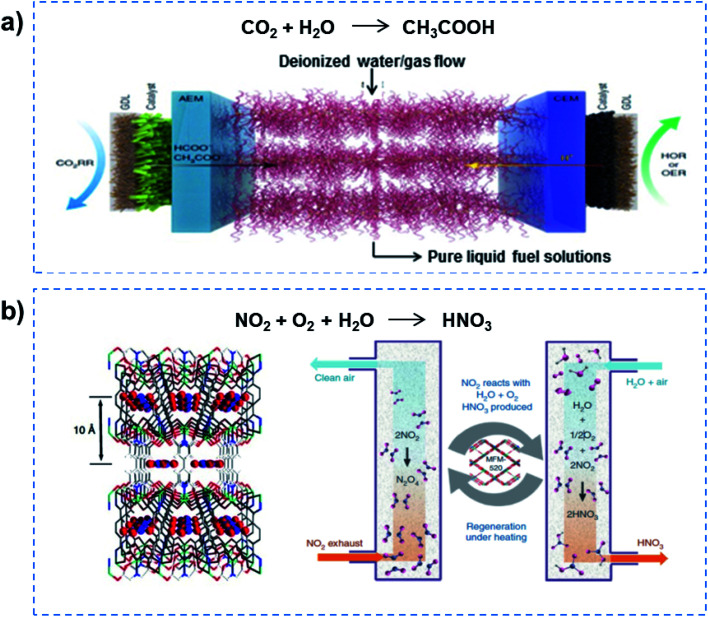
Superfluid-based molecule reactions. (a) Electrocatalytic CO_2_ reduction in solid-state electrolyte containing ion-conducting solid polymers, demonstrating the production of pure HCOOH solutions with high selectivity and activity. Reproduced with permission from ref. 33. Copyright 2019, Nature Publishing Group. (b) A MOF has been utilized to efficiently confine NO_2_ dimer with high adsorption capacity, and realize quantitative conversion into HNO_3_. Reproduced with permission from ref. 35. Copyright 2019, Nature Publishing Group. Superfluid-based molecule reactions can realize excellent performances in terms of high flux and high selectivity.

Further reducing the channel size would allow superfluid-based molecule reactions to proceed, realizing high flux and high selectivity. For example, electrocatalytic CO_2_ reduction in solid-electrolyte devices containing ion-conducting solid polymers has been reported, resulting in continuous production of pure liquid fuel solutions (Fig. 5a).^33^ By using a HCOOH-selective and easily scaled Bi catalyst at the cathode, production of pure HCOOH solutions with concentrations of up to 12 M was demonstrated, also showing 100 h of continuous and stable generation of 0.1 M HCOOH with high selectivity and activity. In addition, a porous solid electrolyte was also demonstrated in use in the direct electrosynthesis of pure aqueous H_2_O_2_ solutions up to 20% by weight and >90% selectivity.^34^ Recently, the metal–organic framework (MOF) MFM-520 has been utilized to efficiently confine a NO_2_ dimer with a high adsorption capacity of 4.2 mmol g^−1^ (Fig. 5b).^35^ The N_2_O_4_ confined inside MFM-520 nanopores was established at the molecular level, which was quantitatively converted into HNO_3_. More importantly, the MFM-520 was fully recovered with no loss of subsequent uptake capacity of NO_2_, indicating continuous clean-up and molecular reaction. Overall, superfluid-based molecule reactions can realize excellent performance in terms of high flux and high selectivity.

## 3. Quantum-confined ion superfluids and QSF ion redox reactions

**Fig. 6:**
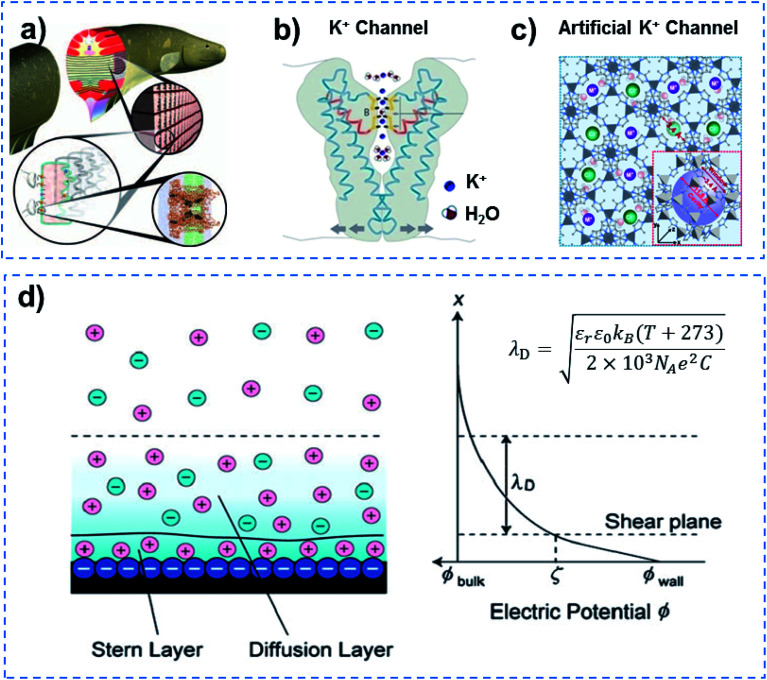
Quantum-confined ion superfluid. (a) Electrocytes in electric eels can generate a high potential of ∼600 V and high current density of 500 A m^−2^ within 20 ms, indicating fast transmission of ions and molecules accurately in the form of a superfluid through Na^+^ and K^+^ channels due to the coherence effect. (b) KcsA K^+^channel of *Streptomyces lividans* with extremely fast ion transport (∼10^7^ ions per s), ultra-low ion resistance and energy consumption, demonstrating the existence of a quantum-confined ion superfluid. Reproduced with permission from ref. 42. Copyright 2004, Wiley-VCH. (c) MOFs with subnanometer pores as an artificial K^+^ channel for ultrafast transport (10^6^–10^8^ ions per s) of alkali metal ions with high selectivity. Reproduced with permission from ref. 44. Copyright 2018, AAAS. (d) Schematic of an electrical double layer (left) and electric potential profile normal to the negatively charged wall (right). A quantum-confined ion superfluid can be formed by decreasing the channel size to a distance of the Debye length (*λ*_D_) for ions. Quantum-confined ion superfluids either in biological or artificial channels show unique characteristics of high flux and low energy consumption.

In addition to quantum-confined molecule superfluids, quantum-confined ion superfluids can also be found in both biological and artificial channels, when the channel size is reduced to the distance of the Debye length (*λ*_D_) for ions. Ultrafast ion and molecule transport are observed in biological ion channels, and approximately 10^7^ ions are allowed to transit in a single channel in 1 s at body temperature.^36^ Electrocytes in electric eels can generate a high potential of ∼600 V and high current density of 500 A m^−2^ within 20 ms (Fig. 6a),^37,38^ indicating the fast transmission of ions and molecules accurately in the form of a superfluid through Na^+^ and K^+^ channels due to the coherence effect.^39^ However, why does an electric eel not kill itself? This means that the resistance of electrocytes in the electric eel is minimal, and they do not generate a lot of heat to kill the animal. Other examples include a NaK nonselective channel, which enables only one fully hydrated Na^+^ ion to be transported through a selective filter.^40^ Similarly, a potassium filter from *Streptomyces lividans* is able to hold an ordered strand containing two K^+^ ions of around 7.5 Å apart and a single water molecule in between (Fig. 6b).^41,42^ Besides this, each calcium channel in calmodulin can also simultaneously bind two Ca^2+^ ions.^43^ In an artificial system, ultrafast ion transport has been reported in MOF channels. The porous ZIF-8 membrane that has an average pore size of ∼0.34 nm demonstrates a high LiCl/RbCl ion selectivity of ∼4.6, and an ultrafast ion transport rate (10^6^–10^8^ ions per s) (Fig. 6c).^44^ UiO-66 MOF channels with pores that are ∼0.6 nm in size show an ultrahigh F^−^ transport rate (10^8^–10^10^ ions per s) and ultrahigh F^−^/Cl^−^ selectivity.^45^ According to the theory of ionics, in liquid phase, counter-ions and co-ions are transported across microchannels based on disordered entropy-driven ion diffusion.^46^ When the channel size decreases to the nanoscale level, specifically close to a distance of two-fold the Debye length (*λ*_D_), the nanochannel is filled with relatively ordered counter-ions in the solution without any co-ions.^47^ Further reducing the channel size to a distance of *λ*_D_ confines the counter-ions in the way of ordered strands, and endows them with ultrafast ion transport in an enthalpy-driven way without energy loss (Fig. 6d). Therefore, quantum-confined ion superfluids either in biological or artificial channels show unique characteristics of high flux and low energy consumption.

**Fig. 7:**
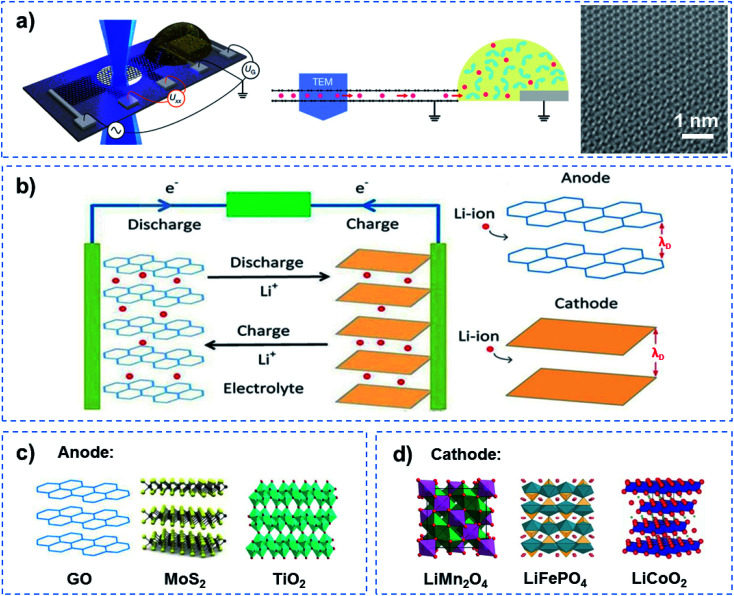
QSF ion redox reactions. (a) Schematic of a device containing bilayer graphene on a Si_3_N_4_-covered Si substrate and a Li-ion electrochemical cell (left), a side view of the device (middle), and a magnified TEM image of the Li crystal (right). This work provided evidence for the superdense ordering of Li in a lattice between two graphene sheets, indicating high energy density and storage capacity for Li batteries. Reproduced with permission from ref. 48. Copyright 2018, Nature Publishing Group. (b) Schematic illustration of a Li ion redox reaction in an anode and cathode when charged and discharged. Ultrafast Li ion transport occurred in a superfluid manner in 2D channels with a distance equivalent to the Debye length (*λ*_D_), resulting in a high charge–discharge rate. Some examples of (c) anode and (d) cathode materials showing a high charge–discharge rate. As for the Li battery, Li redox reactions in the channels of a 2D layered structure have two characteristics, superdense ordering and superfluidity, to realize high energy density and a fast charge–discharge process.

The success of the Li battery demonstrates QSF ion redox reactions in an artificial system. In the charge–discharge process of a Li battery, Li redox reactions in the 2D confined layered structure have two characteristics, superdense ordering and superfluidity, to realize high energy density and fast charge–discharge. In the Li battery, reversible superdense ordering of Li between two graphene sheets has been evidenced by *in situ* transmission electron microscopy (TEM) measurements and density functional theory (DFT) calculations (Fig. 7a).^48^ Li atoms adopt close-packed ordering between the two carbon sheets, resulting in ultrahigh Li storage capacity, which far exceeds that expected from the formation of LiC_6_, the densest configuration known under normal conditions for Li intercalation within bulk graphitic carbon. On the other hand, when charged and discharged, ultrafast transport of Li ions occurs in a superfluid way in the confined 2D channel at a distance of the Debye length (*λ*_D_) for Li ions, resulting in a fast charge–discharge rate (Fig. 7b). Among them, some anodes with excellent performance due to the unique 2D channel structure have been developed (Fig. 7c). For instance, a composite lithium metal anode fabricated by molten Li infusion into a layered rGO film with nanoscale gaps was designed. The anode retains up to ∼3390 mA h g^−1^ of capacity, exhibits low overpotential (∼80 mV at 3 mA cm^−2^) and a flat voltage profile in a carbonate electrolyte.^49^ As an attractive pseudocapacitive electrode material, MoS_2_ nanoparticles with an expanded atomic lamellar structure have been incorporated in a lithium battery, and achieved a maximum power density of 5.3 kW kg^−1^ (with 6 W h kg^−1^ energy density) and a maximum energy density of 37 W h kg^−1^ (with 74 W kg^−1^ power density).^50^ TiO_2_–B nanowires with a length of several hundred nanometers and a width of approximately 10 nm show excellent electron/ion transport properties and reaction kinetics in lithium intercalation, and exhibit an extraordinary rate performance as an anode material for lithium-ion batteries.^51^ On the other hand, cathode materials for high rate performance have also been focused on (Fig. 7d). A LiMn_2_O_4_ nanochain with beads of 100 nm has shown great promise for practical application as a high rate cathode material for Li ion batteries due to its unique subnanochannel structure.^52^ A Li battery with LiFePO_4_ nanoparticles wrapped with a N, S-co-doped graphene composite was shown to realize an ultrahigh rate and long-life for Li ion batteries, owing to the increased Li ion transport rate in its subnanochannels.^53^ Besides this, nanocrystalline LiCoO_2_ with nanosized cell parameters has been adopted as a cathode to achieve high-rate Li-ion intercalation.^54^ It is noteworthy that although John B. Goodenough was awarded the Nobel Prize in Chemistry alongside M. Stanley Whittingham and Akira Yoshino in 2019 for the development of Li-ion batteries, they did not realize that besides high energy density and storage, QSF ion transport and redox reactions in the 2D confined channels of the anode and cathode are the key to a fast charge–discharge process, which is superior to a charge–discharge process dominated by ion diffusion.

**Fig. 8:**
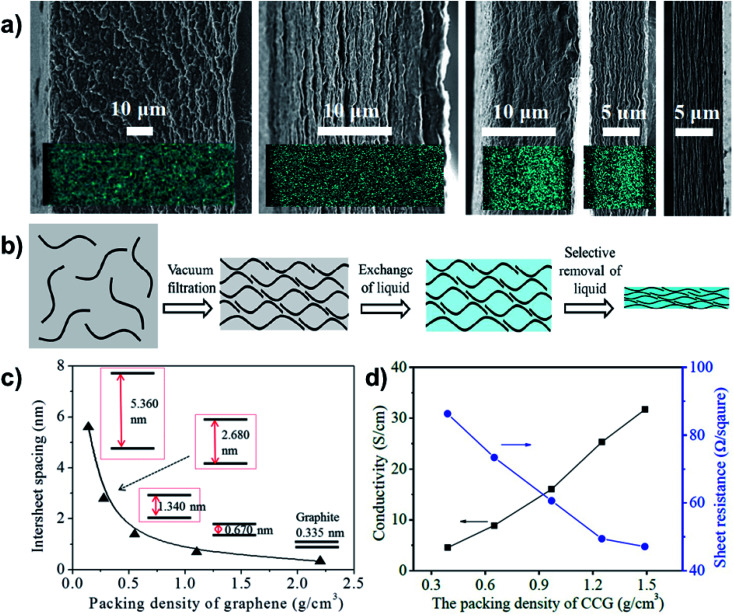
Regulation of the intersheet spacing for QSF ion transport and redox reactions. (a) A porous and densely packed carbon electrode with different intersheet spacings. (b) Schematic illustration of the soft chemistry route to fabricating liquid electrolyte-mediated chemically converted graphene films with different intersheet spacings. (c) Theoretical estimation of the relationship between the intersheet spacing and the packing density of graphene, demonstrating how increasing the packing density reduces the intersheet spacing. (d) Conductivity and sheet resistance of the prepared films with different packing densities, indicating that higher packing density or thinner intersheet spacing improve the conductivity and decrease sheet resistance. Reproduced with permission from ref. 57. Copyright 2013, AAAS. Reducing the intersheet spacing of the 2D layered structure to a distance equivalent to the Debye length (*λ*_D_) leads to high flux and superfluidity of the ions, which are beneficial for the realization of QSF ion transport and redox reactions.

To achieve superdense ordering and superfluidity in QSF ion redox reactions, regulation of the intersheet spacing of a 2D layered structure is of significant importance. As for capacitive energy storage, graphene sheets with subnanometer scale intersheet spacing have been used to form highly compact carbon electrodes with a continuous superfluid-based ion transport network, which achieve ultrahigh volumetric energy densities. The intercalation of Li ions into nanocrystalline layers results in capacitor behaviour, which can be increased as the crystallite size decreases. It has been found that uniquely structured anode and cathode materials can increase capacitance, even at a high rate, especially in a nanoconfined space.^55,56^ Chemically converted graphene hydrogel films with an adaptive pore structure have been fabricated by capillary pressure to adjust intersheet spacing (Fig. 8a).^57^ It is a convenient fabrication route that can be used to achieve a series of pore size films in contrast to traditional techniques (Fig. 8b). The intersheet spacing of graphite is 0.335 nm, while it can vary from 5.360 to 0.670 nm *via* increasing the packing density of graphene (Fig. 8c). Consequently, a higher packing density or thinner intersheet spacing can improve conductivity and decrease sheet resistance (Fig. 8d). Therefore, reducing the intersheet spacing of the 2D layered structure to a distance of the Debye length (*λ*_D_) results in high ion-flux and superfluidity, which are beneficial for the realization of QSF ion transport and redox reactions.

## 4. QSF molecule reactions

**Fig. 9:**
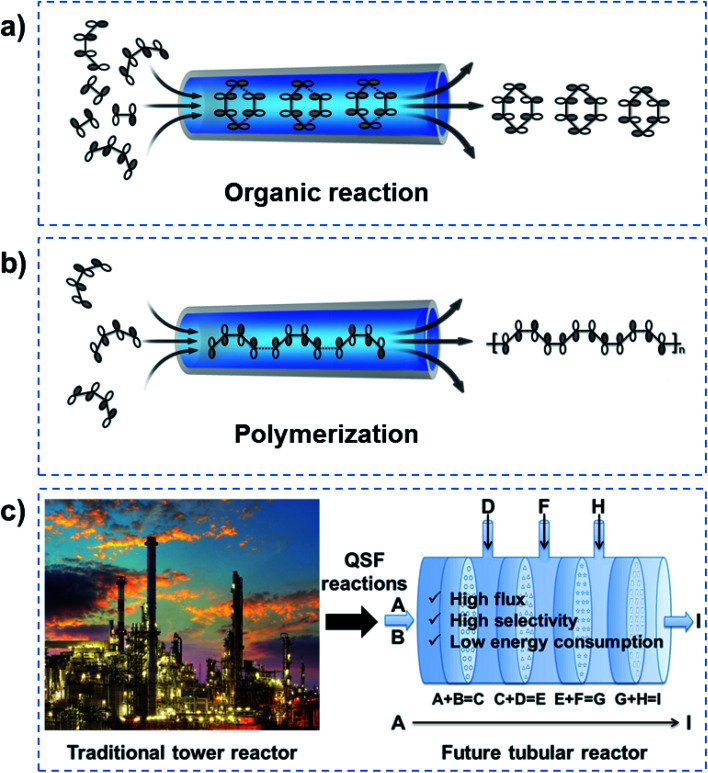
QSF molecule reactions and future chemical engineering. (a) QSF organic reactions. (b) QSF polymerizations. In order to realize QSF reactions in artificial systems, the designs of channels and reactions should be considered. As for the design of channels, the channel size should be designed with a van der Waals equilibrium distance (*r*_0_) for the reactant molecules, to achieve high flux reactions. As for the design of the reactions, through regulating the chemical structure of the channels and the chemical properties of the reactant molecules (polarity, chirality, hydrogen bonding, *etc.*), to arrange the reactants orderly in the channel to satisfy symmetry-matching principles, high performance in terms of 100% selectivity and low reaction activation energy of the reactions can be realized. (c) One-step QSF reactions (A + B = C) can be developed into multi-step QSF reactions (A + B = C, C + D = E, E + F = G, G + H = I, *etc.*), which can be used in future tubular reactors with continuous nanochannel membranes, to achieve high flux, high selectivity and low energy consumption reactions. It is expected that such a tubular reactor will replace the traditional huge tower reactor in future chemical engineering, and bring revolutionary technology to both chemistry and chemical engineering.

In order to realize QSF molecule reactions in an artificial system, on the one hand, reactant molecules should be arranged in order and be able to transform their molecular configuration to satisfy the symmetry-matching principles of the frontier molecular orbital theory, resulting in a lower reaction activation energy of the reactions and achieving 100% selectivity. On the other hand, the channel size should be designed at the van der Waals equilibrium distance (*r*_0_) for molecules, then the reactant fluid can achieve QSF-like ultrafast flow within the channels, resulting in high flux in the reactions. QSF molecule reactions can be further subdivided into QSF organic reactions (Fig. 9a) and QSF polymerization (Fig. 9b), both types exhibiting the characteristics of high flux, low reaction activation energy and 100% selectivity. It is noteworthy that the high flux feature enables the fast adsorption–desorption of reactant molecules on catalysts and reduces the possibility of catalyst deactivation or poisoning to extend the catalyst lifetime.

Based on the above research, one-step QSF reactions (A + B = C) can be developed into multi-step QSF reactions (A + B = C, C + D = E, E + F = G, G + H = I, *etc.*), which can be carried out in a future tubular reactor with continuous nanochannel membranes, to achieve high flux, high selectivity and low energy consumption of the reactions (Fig. 9c). Such tubular reactor is expected to replace the traditional huge tower reactor in future chemical engineering, and would bring revolutionary technology to both chemistry and chemical engineering. It should be noted that heat transfer problems are an important issue in QSF reactions. When there are multi-step QSF reactions, we can design two adjacent reactions where one is exothermic and the other is endothermic, so their heat can offset each other. Heat transfer components can also be designed outside the tubular reactor when heating or cooling is needed. Moreover, there must be a free energy difference to make the QSF reaction devices work. Actually, ions or molecules in a biological system use concentration difference to obtain an energy difference. In an artificial system, we can use concentration difference or pressure difference to obtain an energy difference.

## 5. Summary and outlook

In summary, inspired by enzymatic biosynthesis, we have introduced QSF reactions into artificial systems to achieve high flux, 100% selectivity and low reaction activation energy at room temperature, under atmospheric pressure in an aqueous environment. In order to realize QSF reactions in artificial systems, the design of channels and reactions should be seriously considered. As for the design of channels, according to the molecule reaction or ion redox reaction, we can design the channel size to be the van der Waals equilibrium distance (*r*_0_) for molecules or the Debye length (*λ*_D_) for ions to achieve high flux in the reactions. As for the design of reactions, through regulating the chemical structure of the channels and the chemical properties of the reactant molecules (polarity, chirality, hydrogen bonding, ion interactions, *etc.*), to arrange the reactants orderly in the channel to satisfy symmetry-matching principles, a high performance of 100% selectivity and low reaction activation energy can be realized in the reactions. Additionally, the dynamic wettability of reactant molecules or ions in the channels should be considered in QSF reactions.^58^ In future chemical engineering, based on multi-step QSF reactions, a tubular reactor with continuous nanochannel membranes can be used to achieve high flux, high selectivity and low energy consumption reactions, and is expected to replace the traditional huge tower reactor.

Still, there are many remaining challenges for QSF reactions. For instance, new technology in terms of characterization should be developed to understand the mechanism of QSF reactions. The problems of reaction flow, blockage, and membrane stability should be solved in actual chemical production. A new understanding of the QSF reaction by means of theoretical simulation should be explored. To solve these problems, we can continue to learn from nature and simulate natural systems to achieve better performance.

## Conflicts of interest

There are no conflicts to declare.
